# Cell Selection Game for Densely-Deployed Sensor and Mobile Devices In 5G Networks Integrating Heterogeneous Cells and the Internet of Things

**DOI:** 10.3390/s150924230

**Published:** 2015-09-18

**Authors:** Lusheng Wang, Yamei Wang, Zhizhong Ding, Xiumin Wang

**Affiliations:** School of Computer and Information, Hefei University of Technology, Anhui 230009, China; E-Mails: wangyamei_hfut@sina.com (Y.M.); zzding@mail.ustc.edu.cn (Z.D.); wxiumin@hfut.edu.cn (X.W.)

**Keywords:** Internet of Things, dense sensor networks, heterogeneous cells, cell selection game, inter-cell interference

## Abstract

With the rapid development of wireless networking technologies, the Internet of Things and heterogeneous cellular networks (HCNs) tend to be integrated to form a promising wireless network paradigm for 5G. Hyper-dense sensor and mobile devices will be deployed under the coverage of heterogeneous cells, so that each of them could freely select any available cell covering it and compete for resource with others selecting the same cell, forming a cell selection (CS) game between these devices. Since different types of cells usually share the same portion of the spectrum, devices selecting overlapped cells can experience severe inter-cell interference (ICI). In this article, we study the CS game among a large amount of densely-deployed sensor and mobile devices for their uplink transmissions in a two-tier HCN. ICI is embedded with the traditional congestion game (TCG), forming a congestion game with ICI (CGI) and a congestion game with capacity (CGC). For the three games above, we theoretically find the circular boundaries between the devices selecting the macrocell and those selecting the picocells, indicated by the pure strategy Nash equilibria (PSNE). Meanwhile, through a number of simulations with different picocell radii and different path loss exponents, the collapse of the PSNE impacted by severe ICI (*i.e.*, a large number of picocell devices change their CS preferences to the macrocell) is profoundly revealed, and the collapse points are identified.

## 1. Introduction

After the development of the first four generations, mobile communication systems are encountering new bottlenecks and challenges. On the one hand, many previous hot techniques, such as carrier aggregation, multi-antenna and coordinated multi-point, have been approaching their upper bounds for improving the link-level spectral efficiency [1,2,3]. On the other hand, existing network facilities for mobile communications are constructed outdoors, but in-building-generated phone calls and data traffic are expected to account for more than half of the total volume in the near future [4,5]. To evolve mobile communication systems toward 5G, a new network paradigm has been raised, called heterogeneous cellular networks (HCNs), which is composed of various types of cells deployed complementarily, forming a heterogeneous environment for wireless devices to access [6,7,8]. In HCNs, besides macrocells and microcells covering large-scope areas, picocells and femtocells are deployed to cover hotspots and blind zones, so that seamless and ubiquitous access service can be provided at any time anywhere.

**Figure 1:**
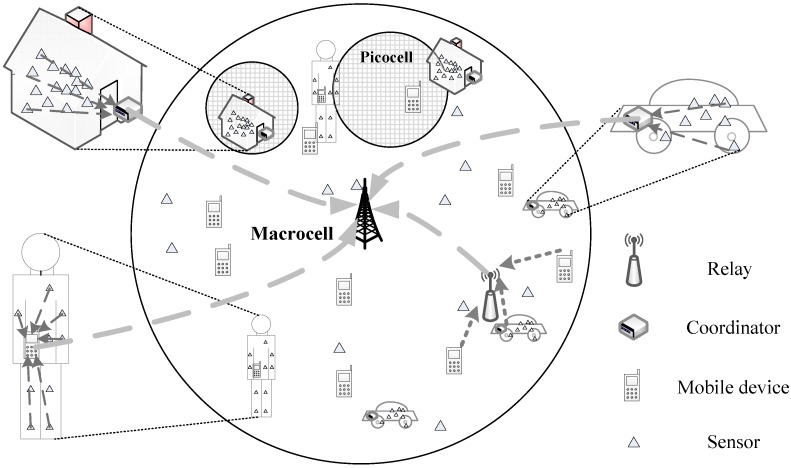
Scenario with multiple picocells and densely-deployed devices in a single macrocell.

In the meantime, the Internet of Things (IoT) is also developing rapidly. Applications, such as smart city [9], Internet of vehicles [10], body area network (BAN) [11] and Internet of bio-nano things [12], were extensively studied, making IoT the most promising concept to integrate the Internet with various domains [13], such as industry, agriculture, healthcare, transportation, markets, education, smart homes, *etc.* Dense deployment will be a new and key feature for future wireless communication systems [14]. Recent studies revealed that IoT and HCNs should integrate to form a new wireless network paradigm for providing real-time and always best connected (ABC) services to both sensor and mobile devices [15,16]. In this network paradigm, mobile devices are able to directly connect to any cell base station (BS) covering it or indirectly connect to a certain BS through a relay. Similarly, sensor devices could also directly transfer data to a BS in the flat networking architecture or indirectly transfer data through a coordinator or relay, such as a mobile device. Figure 1 provides a vision of this network paradigm integrating IoT and HCNs.

Seen from each device’s point of view, it is intelligent to always select the best cell for access while under the coverage of multiple overlapped cells. Decisions are made distributedly and rationally by devices themselves (or their coordinators) based on terminal properties (such as velocity and battery state), application types (such as bandwidth demand and latency demand) and user preferences (such as monetary cost and security), forming a cell selection (CS) game among a large amount of densely-deployed sensor and mobile devices. Meanwhile, seen from the system’s point of view, all of the devices should be associated with their most appropriate cells, so that the whole system performance can be maximized, called CS optimization.

Studies on the CS game are limited in the literature, as shown by the related work summary in Section 2. However, studies on a similar topic, called network selection (NS), also called access technology selection, are extensive, including NS using game theory [17,18,19,20,21]. Various game models have been used, such as the congestion game, evolutionary game and coalition game. In most of these studies, the existence of a pure strategy Nash equilibrium (PSNE) has been proven. Compared to NS, CS is much more complicated due to severe inter-cell interference (ICI) [22,23]. Unlike heterogeneous access technologies (such as cellular networks and WiFi) using orthogonal spectra, different types of cells in HCNs usually share the same portion of the spectrum for their transmissions. Taking the scenario where a picocell is deployed in a macrocell as an example, devices around the picocell, no matter which cell they finally select, will have interference from adjacent devices selecting the other cell. Since the deployment of picocells decreases the average inter-BS distance, HCNs bring about much more severe ICI than homogeneous networks.

To model the CS game with severe ICI, a practical approach is to introduce ICI into the well-known game models for NS. However, this is tough work, because ICI could completely change the features of the payoff functions, hence changing the PSNE in the game. Fortunately, the future network has the key feature of hyper-dense deployment of sensor and mobile devices, guaranteeing the rationality to evaluate the ICI on a given device by taking an average of all of the devices that interfere with it. Meanwhile, no matter whether a device transmits directly or indirectly to a BS, it requires certain resources (*i.e.*, time slot and frequency) and will interfere with near-by devices using the same resource. For the densely-deployed sensors in the near future, coordinators (such as mobiles) will be also densely deployed and be closely surrounded by their sensors. Therefore, the wireless transmission between each coordinator and its BS will also interfere with other transmitters using the same resource.

In this article, we consider a simple, but suitable model, the congestion game [19,20,21], in which the cost function is defined as the congestion that each device undergoes. Then, we add ICI into the payoff function as a weighted summation of congestion and ICI, changing it into a more complicated game. To further evaluate the features of this game, we also consider channel capacity as the payoff function under the conditions of both fractional frequency reuse (FFR) and orthogonal frequency division (OFD). Three theorems are proven for PSNE in the above games. In the end, extensive simulations show the impact of ICI on the PSNE of these CS games.

The contribution of this study is two-fold. On the one hand, we theoretically find the circular boundary between the devices selecting the macrocell and those selecting picocells, *i.e.*, the PSNE of the CS game among densely-deployed sensor and mobile devices. Three theorems are proven, and their correctness is verified by simulations with the ICI formulated by integrals. On the other hand, we systematically analyze the severe impact of ICI on the PSNE by extensive simulations. The collapse of the PSNE (*i.e.*, a large number of picocell devices changes their CS preferences to the macrocell in the PSNE) and the degradation of the system performance are clearly revealed, which is quite helpful for the future CS scheme design in the HCN and IoT integrated environment.

The remainder of this article is organized as follows. In Section 2, a summary of CS studies is provided. In Section 3, we describe the system model. In Section 4, we theoretically analyze the PSNE of three game models. In Section 5, extensive simulations are performed to demonstrate the correctness of the theorems and the impact of ICI on the CS games and the collapse points. Finally, in Section 6, we draw the conclusions.

## 2. Related Works

Studies on CS can be traced back to the era of homogeneous networks, such as the work by Hanly [24], Zhang and Letaief [25], Sang *et al.* [26], *etc.* The issue becomes much more complicated when it is in a heterogeneous environment due to the multi-attribute feature of various access technologies and cells. For a heterogeneous wireless network environment composed of WiFi, cellular access technology, personal area networks, *etc.*, NS has been widely studied [17]. Optimization techniques, such as multi-attribute decision making, fuzzy logic and utility theory, have been used for deciding the best network to access. Game theory was also used to study the competition between users for accessing these networks [18].

However, the above two research directions, *i.e.*, CS in homogeneous networks and NS in heterogeneous networks, did not cover the studies on the CS in HCNs, which attracted much attention in recent years. Cells in HCNs not only have a multi-attribute feature, but also suffer from ICI, which brings in new challenges for a CS decision. Existing studies can be divided into two categories, *i.e.*, optimization solutions, as summarized in Table 1, and game theory solutions, as summarized in Table 2. CS optimization was studied from the system-level point of view, *i.e.*, the performance optimization of the whole heterogeneous environment. By contrast, the CS game was studied from the user-level point of view, where each game player rationally aims to improve his/her own benefits. For the two tables, we summarize mainly the mathematical methodologies used in these papers. For simulation studies based on some widely-used parameters, such as reference signal receiving power (RSRP), reference signal receiving quality (RSRQ) and signal-to-interference-plus-noise ratio (SINR), we also point out the key parameters used in their studies. For theoretical studies combining CS with other techniques, such as ICI coordination and resource allocation (RA), we point out that they are joint optimizations or games combining multiple techniques. Considering the scope of this study, we further describe in detail the related studies on CS games in HCNs as follows.

**Table 1:** Summary of CS optimization of the entire system.

Category	Reference	Features
Range expansion	[27]	SINR
	[28]	Almost blank subframe (ABS), expected user data rate
	[29]	Proportional fairness
	[30]	Bias design
	[31]	Asymmetric downlink/uplink ICI coordination
	[32]	ABS, user association probability, throughput
Association	[33]	Downlink max-min sum rate, convex
	[34]	Total capacity and coverage gains across network
	[35]	Outage probability, average ergodic rate, minimum user throughput
	[36]	Load balancing, gradient descent method, online algorithm
	[37]	Load-aware, best biasing, convex
	[38]	Network capacity, load balancing, pricing based
	[39]	Generalized algebraic framework, load balancing, greedy
	[40]	Energy efficiency, spectral efficiency, cognitive radio
	[41]	Load balancing, distributed near-optimal solution
	[42]	Network utility maximization, proportional fairness, pricing based
Joint CS-RA	[43]	Water filling, bisection search, sum-power and sum-rate constraint
	[44]	Benders’ decomposition, non-convex BS association, power control
	[45]	Joint user association, channel assignment, beamforming, power control
	[46]	Orthogonal, co-channel, partially shared, non-convex integer program
	[47]	Maximum capacity, max-min fairness
Fast CS	[48]	Gain in throughput
	[49]	SINR
Performance evaluation	[50]	SINR, capacity
	[51]	SINR, RSRP, RSRQ
	[52]	RSRP, range expansion with static/adaptive offset or with ABS
	[53]	Path loss, SINR, capacity
Others	[54]	Knapsack, assignment problem
	[55]	Maximum expected bitrate
	[56]	Maximum ergodic capacity
	[57]	Percentage of the total earned profit
	[58]	Aggregate energy consumption

Lin *et al.* [59] provided a general form of the CS game and proved the existence of PSNE. Yuan *et al.* [60] studied the case where a femtocell changes from closed access to open access, so that nearby users could access it instead of accessing the macrocell, which decreased uplink ICI from these users to the femtocell. A Stackelberg game was considered between the femtocell BS as the leader and the macrocell user as the follower to improve their performance by establishing links between them. Moon and Cho [61] proposed a CS algorithm based on competition among a group of users, each of which decided the probability of choosing a microcell as its serving cell. Haddad *et al.* [62] considered a Bayesian game and showed by means of a Stackelberg formulation that the operator could optimize its global utility while the end-users maximized their individual utilities. Liu *et al.* [63] proposed a Nash bargaining solution, which formulated the user association optimization as a Nash bargaining problem, so as to maximize the sum data rate related utility of all users in the overall system while guaranteeing the user’s minimal data rate and considering user fairness. Gao *et al.* [64] modeled the game on two levels, *i.e.*, inter-cell CS game and intra-cell RA game, proved that the CS game had mixed strategy Nash equilibrium (MSNE) and proposed a distributed algorithm that converged to MSNE.

**Table 2:** Summary of the CS game between devices.

Category	Reference	Features
General form	[59]	PSNE existence proof
Evolutionary	[65]	Reinforcement learning to search for evolutionary equilibrium
Stackelberg	[60]	Femtocell changes from closed to open access against uplink ICI
Bargaining	[63]	ICI coordination, Nash bargaining solution
Bayesian	[62]	Maximum throughput, Stackelberg formulation
Joint CS-RA	[64]	MSNE existence prove, distributed algorithm converges to MSNE
Competition	[61]	Probability to select certain cell
Congestion	[66]	Prove of PSNE under inter-cell interference
Learning based	[67]	Minimize the number of outages
	[68]	Distributed decision making toward PSNE
	[69]	Predict best cell during handover decision

Some studies considered intelligent learning algorithms to search for the PSNE. Feng *et al.* [65] proposed an evolutionary game model for CS in a two-tier femto/macro scenario, and the reinforcement learning algorithm was used to search for the evolutionary equilibrium. Tan *et al.* [68] studied the CS problem in a two-tier femtocell network. To achieve PSNE without a centralized controller, the Q-learning algorithm was used to help distributed individual users adapt and make decisions independently. Dhahri and Ohtsuki [69] proposed a Q-learning-based CS scheme, which predicted the best cell by the Q-learning algorithm during the handover procedure, so it improved the system capacity, avoided unnecessary handovers and decreased signaling cost. Kudo and Ohtsuki [67] proposed a scheme to select a cell by the Q-learning algorithm to minimize the number of outages from its past experience independently. Liao *et al.* [66] tried to embed ICI into the CS congestion game in a scenario with one macrocell and one picocell. PSNE was analyzed in both theoretical and simulative ways. In this article, for densely-deployed sensor and mobile devices in HCNs with multiple picocells, we work on the analysis of PSNE collapse toward the macrocell impacted by severe ICI and the corresponding degradation of system performance, which is not revealed by any of the above studies.

## 3. System Model

We consider the case where a single macrocell $B\;S_{0}$ and a set of open access picocells $\left\{B\;S_{i}\;|i\;=\;1,\ldots ,M\right\}$ coexist and overlap with one another. We ignore the macrocells around this single macrocell, because ICI between heterogeneous cells obviously has a higher magnitude than ICI between homogeneous cells, so this simplification does not affect the results much. There are in total $N_{0}$ densely-deployed sensor and mobile devices in the scenario, given by $D\;V_{All}=\left\{D\;V_{j}|j=1,\ldots ,N_{0}\right\}$. Note that the assumption of the dense deployment of the devices guarantees the rationality to evaluate the ICI on a given device by taking an average of all of the devices that interfere with it, as analyzed in Section 4.2 later. The cell radius of $B\;S_{i}$ is denoted by $R_{i}$, and the number of devices under the coverage of $B\;S_{i}$ is given by $N_{i}$. We assume that all of the devices are uniformly distributed within the disc coverage area of $B\;S_{0}$, so the density of devices is given by $\rho =N_{0}/\pi R_{0}^{2}$. $N_{i}=N_{0}R_{i}^{2}/R_{0}^{2}$, ∀i∈{1,…,M}. Main notations are summarized in Table 3. Without a specific description, notations follow the rule that, the subscript i=0 represents the macrocell and i∈{1,…,M} represents the *i*-th picocell.

**Table 3:** Main notations.

$B\;S_{i}$	BS of cell *i*
$D\;V_{i}$	Devices selecting $B\;S_{i}$
$D\;V_{All}$	All of the devices within the coverage of the macrocell disc area
$D\;V_{j}$	The *j*-th device in a set of devices
$N_{i}$	Number of devices within the coverage of cell *i*
$R_{i}$	Radius of the disc area covered by $B\;S_{i}$
$P_{i}$	Uplink transmission power of the devices to $B\;S_{i}$
$B_{i}$	Total available bandwidth for cell *i*
$I_{ij}$	Average ICI from the devices in cell $B\;S_{i}$ to $B\;S_{j}$
$n_{i}$	Number of devices selecting $B\;S_{i}$
$D_{ij}$	Distance between $B\;S_{i}$ and $B\;S_{j}$
$d_{ij}$	Distance between $B\;S_{i}$ and $D\;V_{j}$
$r_{i}$	Radius of the disc area formed by the devices selecting cell *i*
$p_{j}$	Pure strategy of $D\;V_{j}$
$U_{j}$ / $C_{j}$	Utility/cost function of $D\;V_{j}$

**Figure 2:**
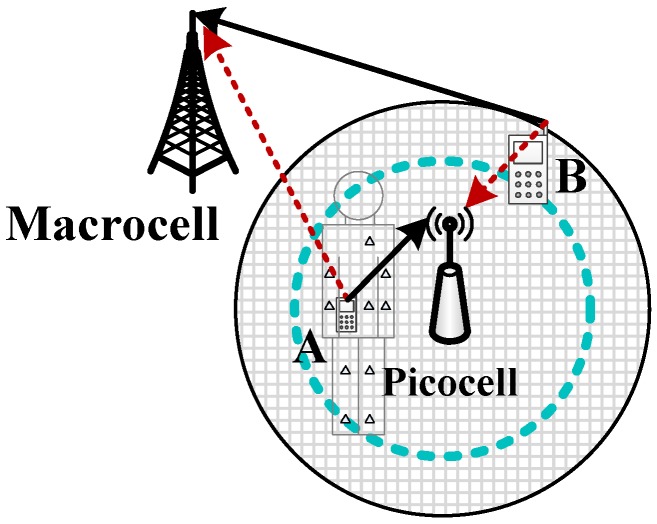
System model. Each picocell holds a circular boundary (*i.e.*, the azure dashed circle) indicating the PSNE. Device A is inside the boundary, so it transmits to the picocell and interferes with the macrocell. Although Device B is within the edge of the picocell, it is outside of the dashed circle, so it selects the macrocell and interferes with the picocell.

Devices selecting $B\;S_{i},i\in \left\{1,\ldots ,M\right\}$ are given by $D\;V_{i}$. Data can be transmitted directly between the BS and a device or indirectly through a coordinator (or relay). For indirect transmission through a coordinator, we assume that the link between the BS and the coordinator is wireless, so it is identical to the case when the coordinator itself transmits to the BS. Meanwhile, transmissions between sensors and their coordinators are on unlicensed frequency bands with extremely low transmission power, which do not cause ICI and do not even occupy resources in licensed bands. In a word, direct and indirect connections can be modeled identically for evaluating ICI in the CS game. We assume that all of the devices are identical for their traffic type, terminal property and user preference, so it is reasonable to assume that $D\;V_{i}$ are the $n_{i}$ devices closest to $B\;S_{i}$, forming a disc area with radius $r_{i}$, and $n_{i}=N_{0}r_{i}^{2}/R_{0}^{2}$, as demonstrated by the azure circle in Figure 2. If the above features of various devices are not identical in reality, a function combining these features is used to represent the unified tendency for selecting each cell, which can be considered as a virtual distance when mapping into our system model. Therefore, the above assumption still holds when those features are not identical, while the $n_{i}$ devices are the closest to $B\;S_{i}$ in terms of the virtual distance (corresponding to the $n_{i}$ devices with the highest tendency to $B\;S_{i}$). The objective of this study is to identify $n_{i}$ corresponding to the PSNE of the game below:

(1) $$G=\{D\;V_{All},{\left(p_{j}\right)}_{j\in \{1,\ldots ,N_{0}\}},{\left(U_{j}\right)}_{j\in \{1,\ldots ,N_{0}\}}\}$$

where $D\;V_{All}$ are the players, $p=\{p_{j}|j=1,\ldots ,N_{0}\}$ are the pure strategies used by the players, each player’s strategy is chosen from the pure strategy set $\left\{B\;S_{i}|i=0,1,\ldots ,M\right\}$ and ${\left(U_{j}\right)}_{j\in \{1,\ldots ,N_{0}\}}$ are the players’ payoff functions, which are assumed to be identical and related to the number of devices selecting the same cell, the positions of devices and ICI. Equation (1) is a class of non-cooperative game, called the congestion game, as long as $\forall j\in \{1,\ldots ,N_{0}\}$; $U_{j}$ is a utility function of the number of devices selecting the same BS with a monotonically non-increasing feature (or non-decreasing feature if congestion $C_{j}$ is used as a cost function).

Given pure strategies p, PSNE is defined as [70]:

**Definition 1.**  *A set of pure strategies,* $p^{*}$ *is called a PSNE if* $\forall j\in \{1,\ldots ,N_{0}\}$ *and* $\forall p_{j}\in p$,

(2) $$U_{j}\left(p_{j}^{*},p_{-j}^{*}\right)\geq U_{j}\left(p_{j},p_{-j}^{*}\right)$$

Based on the assumptions above, given a set $n^{*}=\left\{n_{i}|i=1,\ldots ,M\right\}$, $p^{*}$ is fixed, so the definition of PSNE could be changed into the following:

**Definition 2.**  $n^{*}$ *is said to be a PSNE if its corresponding* $p^{*}$ *satisfies Definition 1.*

## 4. Cell Selection Games

Since picocells usually have a small coverage and are deployed at hotspots for complementation, we assume that their coverage areas are independent of one another. In this scenario, we are interested in $n_{i}^{*}$, the number of devices selecting $B\;S_{i}$ in the PSNE.

### 4.1. Traditional Congestion Game

We use the traditional congestion game (TCG) to model the CS problem with multiple picocells. In this model, congestion of a device is proportional to the total number of devices selecting the same BS, so the cost of $D\;V_{k}$ selecting the macrocell $B\;S_{0}$ and the cost of $D\;V_{l}$ selecting picocell $B\;S_{i},i\in \left\{1,\ldots ,M\right\}$ can be the inverse of the amount of resource obtained by $D\;V_{k}$ and $D\;V_{l}$, written as:

(3) $$C_{k}\left(B\;S_{i},p_{-k}\right)=\frac{n_{i}}{B_{i}}$$

(4) $$C_{l}\left(B\;S_{0},p_{-l}\right)=\frac{N_{0}-\sum_{\alpha }n_{\alpha }}{B_{0}}$$

where $B_{0}$ and $B_{i}$ represent the total uplink bandwidth for $B\;S_{0}$ and $B\;S_{i}$, respectively.

If $D\;V_{k}$ changes the CS preference to $B\;S_{0}$, its cost becomes:

(5) $$C_{k}\left(B\;S_{0},p_{-k}\right)=\frac{N_{0}-\sum_{\alpha }n_{\alpha }+1}{B_{0}}$$

Similarly, if $D\;V_{l}$ changes the CS preference to $B\;S_{i}$, its cost becomes:

(6) $$C_{l}\left(B\;S_{i},p_{-l}\right)=\frac{n_{i}+1}{B_{i}}$$

**Theorem 1.**  *For TCG,* ∀i∈{1,…,M}*, define:*

(7) $$g_{1}\left(n_{i}\right)=n_{i}+\frac{B_{i}}{B_{0}+B_{i}}\sum_{\alpha \neq i}n_{\alpha }$$

(8) $$A_{1,i}=\frac{B_{i}\left(N_{0}+1\right)}{B_{0}+B_{i}}$$

*(1) if* $g_{1}\left(N_{i}\right)<A_{1,i}$*,* $n_{i}^{*}=N_{i}$ *forms the PSNE;*

*(2) if* $g_{1}\left(1\right)>A_{1,i}$*,* $n_{i}^{*}=0$ *forms the PSNE;*

*(3) otherwise,* $n_{i}^{*}$ *satisfying* $A_{1,i}-1\leq g_{1}\left(n_{i}^{*}\right)\leq A_{1,i}$ *forms the PSNE.*

**Proof.**  Based on Definition 1, we rewrite the conditions that $n_{i}$ forms the PSNE as, ∀i∈{1,…,M}:

(9) $$C_{k}\left(B\;S_{i},p_{-k}\right)\leq C_{k}\left(B\;S_{0},p_{-k}\right)$$

(10) $$C_{l}\left(B\;S_{0},p_{-l}\right)\leq C_{l}\left(B\;S_{i},p_{-l}\right)$$

Equation (9) and Equation (10) separately indicate that no device selecting the picocell $B\;S_{i}$ and the macrocell $B\;S_{0}$ feels like changing the CS preference.

Taking Equations (3)–Equation (6) into (9) and (10), we get:

(11) $$A_{1,i}-1\leq g_{1}\left(n_{i}^{*}\right)\leq A_{1,i}$$

**Figure 3:**
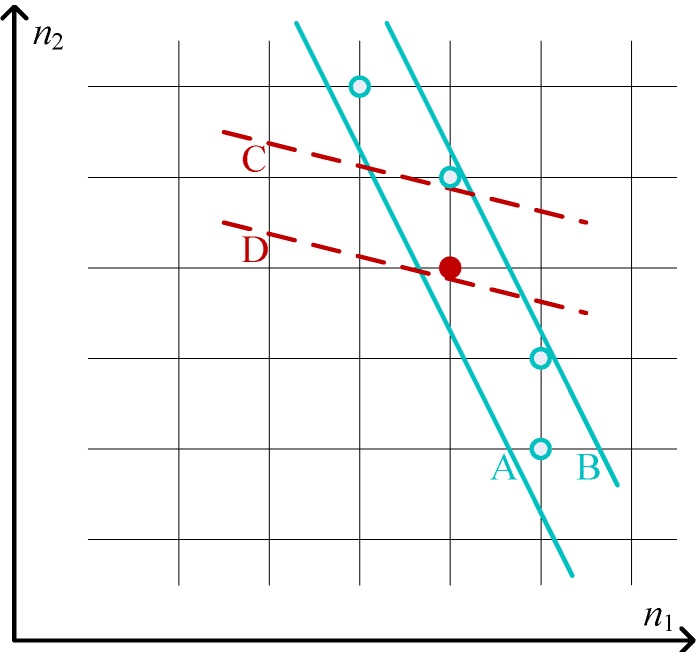
Two-dimensional case of the traditional congestion game (TCG) for the proof of Theorem 1.

To prove the existence of a PSNE based on Equation (11), we consider firstly the two-dimensional case with $n_{1}$ and $n_{2}$ shown in Figure 3. For $A_{1,1}-1\leq g_{1}\left(n_{1}\right)\leq A_{1,1}$, the integer points between the two parallel solid lines fit it. Since the distance between Point A and B is one and the slope coefficient of the two solid lines corresponding to the above inequation is less than −1, given each $n_{1}$, there is at least one integer solution for $n_{2}$.

Meanwhile, since the slope coefficient of the two parallel dashed lines for $A_{1,2}-1\leq g_{1}\left(n_{2}\right)\leq A_{1,2}$ is in (-1,0) and the distance between Point C and D is one, at least one integer solution between the two solid lines fits for the above inequation, *i.e.*, at least one solution can be found for Equation (11) with i=1,2.

Applying mathematical induction, $\forall \{n_{1},n_{2},\ldots ,n_{k}\}$ as a *k*-dimensional point in the *k*-dimensional case, there is at least one integer solution for $n_{k+1}$. Since the two parallel dashed lines in the (k+1)-th dimension have a slope coefficient in (-1,0), at least one integer solution fits for $A_{1,k+1}-1\leq g_{1}\left(n_{k+1}\right)\leq A_{1,k+1}$, indicating the existence of a PSNE.

Since $B\;S_{i}$ covers $N_{i}$ devices, if $g_{1}\left(N_{i}\right)<A_{1,i}$, the PSNE corresponds to $n_{i}^{*}=N_{i}$, indicating that all of the devices covered by $B\;S_{i}$ select it. If $g_{1}\left(1\right)>A_{1,i}$, the PSNE corresponds to $n_{i}^{*}=0$. Otherwise, $n_{i}^{*}$ satisfying Equation (11) forms the PSNE.

To sum up, Theorem 1 indicates the numbers of devices selecting different picocells in the PSNE. $g_{1}\left(n_{i}\right)$ in Theorem 1 is a combination of the variables representing the numbers of devices selecting different picocells. When this combined value satisfies Theorem 1, a PSNE is reached.

### 4.2. Congestion Game with ICI

The deployment of heterogeneous cells decreases the inter-BS distance on average, which severely increases ICI. The congestion model in the previous subsection is simple, but it does not take ICI into account. In this subsection, we consider the game with a cost function combining congestion and ICI linearly, called the congestion game with ICI (CGI).

Since the device that interferes with the device $D\;V_{j}$ is highly related to the RA strategy and can be any device selecting adjacent cells, the estimation of the ICI for a general CS problem is quite difficult. The study in this article focuses on the scenario with densely-deployed devices, where the average ICI from devices in other cells can be used to represent the ICI that $D\;V_{j}$ undergoes. Therefore, a device’s uplink transmission to the picocell $B\;S_{i}$ undergoes an average ICI from devices using the macrocell $B\;S_{0}$, which is a function of $n_{i}$, given by:

(12) $$I_{0i}\left(n_{i}\right)=\frac{GP_{0}}{N_{0}-\sum_{\alpha }n_{\alpha }}\left(\sum_{j\in D\;V_{All}}\frac{1}{d_{ij}^{\gamma }}-\sum_{\alpha }\sum_{j\in D\;V_{\alpha }}\frac{1}{d_{\alpha j}^{\gamma }}\right)$$

where $P_{0}$ is the transmission power of the devices to $B\;S_{0}$, γ is the path loss exponent, $G=G_{t}G_{r}\lambda^{2}/4\pi^{2}$ is a constant related to transmission antenna gain $G_{t}$, reception antenna gain $G_{r}$ and wavelength *λ*. Note that we do not consider shadowing or multipath fading due to the fact that our model and analysis are statistical for densely-deployed sensor and mobile devices, so the instantaneous signal strength of a particular device does not affect the integral results much.

Compared to $R_{0}$, the radius of the picocell $R_{i}$ is small, so we ignore the position difference of the devices in it when calculating the average interference to $B\;S_{0}$, given by:

(13) $$I_{i0}=\frac{GP_{i}}{D_{0i}^{\gamma }}$$

where $P_{i}$ is the transmission power of the devices to $B\;S_{i}$.

**Figure 4:**
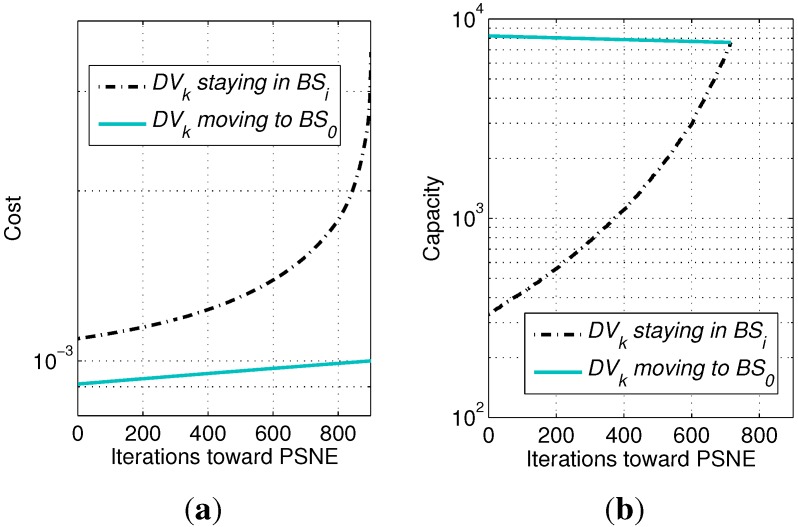
Monotonicity of payoff functions in Theorems 2 and 3. Obtained by setting $B_{i}=2/3B_{0}$, $R_{i}=300$ m, $w=3\times 10^{6}$ and other parameters commonly configured as in Section 5, for a scenario with one picocell located within the macrocell. Curves are obtained by searching for the PSNE, so the number of devices selecting the picocell gradually decreases during iterations. (**a**) Cost in Theorem 2; (**b**) capacity in Theorem 3.

Therefore, the cost of $D\;V_{k}$ using $B\;S_{i}$ is given by:

(14) $$C_{k}\left(B\;S_{i},p_{-k}\right)=\frac{n_{i}}{B_{i}}+wI_{0i}\left(n_{i}\right)$$

where *w* is the weight of ICI, which is required, because ICI and congestion have different magnitudes and are difficult to normalize. *w* should not be too large, otherwise it changes the monotonicity of Equation (14), and the PSNE will collapse to the macrocell without any equilibrium between $N_{i}$ and zero. Figure 4a shows that, once a picocell device decides to select the macrocell, it leads to a much faster increment of the cost for other devices still preferring the picocell. Therefore, devices will change their preferences one by one until an equilibrium is reached where only a few devices or even no device still prefers the picocell.

Similarly, the cost of $D\;V_{l}$ using $B\;S_{0}$ is given by:

(15) $$C_{l}\left(B\;S_{0},p_{-l}\right)=\frac{N_{0}-\sum_{\alpha }n_{\alpha }}{B_{0}}+wI_{i0}$$

After $D\;V_{k}$ changes the CS preference to $B\;S_{0}$, its cost becomes:

(16) $$C_{k}\left(B\;S_{0},p_{-k}\right)=\frac{N_{0}-\sum_{\alpha }n_{\alpha }+1}{B_{0}}+wI_{i0}$$

Since ICI is related to the positions of devices, we assume that the device that has the strongest willingness to change the CS preference to $B\;S_{i}$ is the one closest to the circular boundary. Therefore, after $D\;V_{l}$ changes the CS preference to $B\;S_{i}$, its cost becomes:

(17) $$C_{l}\left(B\;S_{i},p_{-l}\right)=\frac{n_{i}+1}{B_{i}}+wI_{0i}\left(n_{i}+1\right)$$

**Theorem 2.**  *For CGI, define:*

(18) $$g_{2}\left(n_{i}\right)=n_{i}+\frac{B_{i}}{B_{i}+B_{0}}\sum_{\alpha \neq i}n_{\alpha }+\frac{B_{i}B_{0}}{B_{i}+B_{0}}wI_{0i}\left(n_{i}\right)$$

(19) $$A_{2,i}=\frac{B_{i}}{B_{i}+B_{0}}\left(N_{0}+1+B_{0}wI_{i0}\right)$$

*(1) if* $g_{2}\left(N_{i}\right)<A_{2,i}$*,* $n_{i}^{*}=N_{i}$ *forms the PSNE;*

*(2) if* $g_{2}\left(1\right)>A_{2,i}$*,* $n_{i}^{*}=0$ *forms the PSNE;*

*(3) otherwise,* $n_{i}^{*}$ *satisfying* $g_{2}\left(n_{i}^{*}\right)\leq A_{2,i}\leq g_{2}\left(n_{i}^{*}+1\right)$ *forms the PSNE.*

**Proof.**  Based on Definition 1, we obtain the conditions for the PSNE of CGI as follows:

(20) $$C_{k}\left(B\;S_{i},p_{-k}^{*}\right)\leq C_{k}\left(B\;S_{0},p_{-k}^{*}\right)$$

(21) $$C_{l}\left(B\;S_{0},p_{-l}^{*}\right)\leq C_{l}\left(B\;S_{i},p_{-l}^{*}\right)$$

Equation (20) and Equation (21) indicate separately that no device selecting $B\;S_{i}$ and $B\;S_{0}$ feels like changing the CS preference.

Taking Equation (14)–Equation (17) into (20) and (21), we get:

(22) $$g_{2}\left(n_{i}^{*}\right)\leq A_{2,i}\leq g_{2}\left(n_{i}^{*}+1\right)$$

To sum up,

(1) If $g_{2}\left(N_{i}\right)<A_{2,i}$, all of the devices under the coverage of $B\;S_{i}$ select it, and $n_{i}^{*}=N_{i}$ forms the PSNE.

(2) If $g_{2}\left(1\right)>A_{2,i}$, all of the devices under the coverage of $B\;S_{i}$ select $B\;S_{0}$, and $n_{i}^{*}=0$ forms the PSNE. This case cannot be indicated by Equation (22) because the integral for $I_{0i}\left(n_{i}\right)$ is equivalent to an extremely large number of devices deployed in the area. In such a case, there is absolutely one device collocated with $B\;S_{i}$, leading to infinity for $g_{2}\left(0\right)$.

(3) Otherwise, $n_{i}^{*}$ satisfying Equation (22) forms the PSNE. Since $I_{0i}\left(n_{i}\right)$ is a decreasing function with quite a small value, as long as *w* is kept below a certain threshold, the gap between $g_{2}\left(n_{i}^{*}\right)$ and $g_{2}\left(n_{i}^{*}+1\right)$ is smaller than one, so there is always at least one $n_{i}^{*}$ satisfying it.

### 4.3. Congestion Game with Capacity

The game model in the previous subsection focuses on the linear combination of congestion and ICI, so the precision of such a game depends on the weight of ICI. We look for a more realistic payoff function to represent the impact of ICI, so channel capacity is considered in this subsection. We first consider the case where FFR for ICI coordination is adopted by picocells, called the congestion game with capacity under the condition of FFR (CGC-FFR), so the utility functions can be written as follows.

The channel capacity of $D\;V_{k}$ selecting the picocell $B\;S_{i}$ is:

(23) $$U_{k}\left(B\;S_{i},p_{-k}\right)=\frac{B_{i}}{n_{i}}\log_{2}\left(1+\frac{GP_{i}/d_{ik}^{\gamma }}{\sigma^{2}+I_{0i}\left(n_{i}\right)}\right)$$

where $\sigma^{2}$ represents the variance of additive white Gaussian noise (AWGN). Due to the fact that the device on the edge of the circular boundary has the strongest willingness to change the CS preference, $d_{ik}$ is used as the distance between $D\;V_{k}$ and $B\;S_{i}$. If $D\;V_{k}$ changes preference to the macrocell $B\;S_{0}$, its channel capacity becomes:

(24) $$U_{k}\left(B\;S_{0},p_{-k}\right)=\frac{B_{0}}{N_{0}-\sum_{\alpha }n_{\alpha }+1}\log_{2}\left(1+\frac{GP_{0}/D_{0i}^{\gamma }}{\sigma^{2}+I_{i0}}\right)$$

Figure 4b shows the monotonicity of the above two payoff functions. The curves show that, when the devices gradually change their preferences to the macrocell, the other devices’ willingness (*i.e.*, the capacity) to select the picocell increases, while their willingness to select the macrocell decreases. When the two curves meet after 716 iterations, an equilibrium is reached.

Similarly, the channel capacity of $D\;V_{l}$ selecting $B\;S_{0}$ is:

(25) $$U_{l}\left(B\;S_{0},p_{-l}\right)=\frac{B_{0}}{N_{0}-\sum_{\alpha }n_{\alpha }}\log_{2}\left(1+\frac{GP_{0}/D_{0i}^{\gamma }}{\sigma^{2}+I_{i0}}\right)$$

If $D\;V_{l}$ hands over to $B\;S_{i}$, its channel capacity is:

(26) $$U_{l}\left(B\;S_{i},p_{-l}\right)=\frac{B_{i}}{n_{i}+1}\log_{2}\left(1+\frac{GP_{i}/d_{il}^{\gamma }}{\sigma^{2}+I_{0i}\left(n_{i}+1\right)}\right).$$

**Theorem 3.**  *For CGC-FFR, define:*

(27) $$g_{3}\left(n_{i}\right)=\log_{2}{\left(1+\frac{GP_{i}{\left[N_{0}/\left(n_{i}R_{0}^{2}\right)\right]}^{\gamma /2}}{\sigma^{2}+I_{0i}\left(n_{i}\right)}\right)}^{\frac{N_{0}-\sum_{\alpha }n_{\alpha }+1}{n_{i}}}$$

(28) $$A_{3,i}=\log_{2}{\left(1+\frac{GP_{0}/D_{0i}^{\gamma }}{\sigma^{2}+I_{i0}}\right)}^{\frac{B_{0}}{B_{i}}}$$

*(1) if* $g_{3}\left(N_{i}\right)>A_{3,i}$*,* $n_{i}^{*}=N_{i}$ *forms the PSNE;*

*(2) if* $g_{3}\left(1\right)<A_{3,i}$*,* $n_{i}^{*}=0$ *forms the PSNE;*

*(3) otherwise,* $n_{i}^{*}$ *satisfying* $g_{3}\left(n_{i}^{*}+1\right)\leq A_{3,i}\leq g_{3}\left(n_{i}^{*}\right)$ *forms the PSNE.*

**Proof.**  Based on Definition 1, we obtain the conditions for the PSNE of CGC-FFR as follows:

(29) $$U_{k}\left(B\;S_{i},p_{-k}^{*}\right)\geq U_{k}\left(B\;S_{0},p_{-k}^{*}\right)$$

(30) $$U_{l}\left(B\;S_{0},p_{-l}^{*}\right)\geq U_{l}\left(B\;S_{i},p_{-l}^{*}\right)$$

Since all of the devices are assumed identical, the device most preferably changing its preference is the one on the edge of the blue circle in Figure 2, so:

(31) $$d_{ik}^{\gamma }={\left(\frac{R_{0}^{2}n_{i}^{*}}{N_{0}}\right)}^{\gamma /2}$$

(32) $$d_{il}^{\gamma }={\left[\frac{R_{0}^{2}\left(n_{i}^{*}+1\right)}{N_{0}}\right]}^{\gamma /2}$$

Taking Equation (23)–Equation (26), (31) and (32) into (29) and (30), we get:

(33) $$g_{3}\left(n_{i}^{*}+1\right)\leq A_{3,i}\leq g_{3}\left(n_{i}^{*}\right)$$

To sum up,

(1) If $g_{3}\left(N_{1}\right)\geq A_{3,i}$, all of the devices under the coverage of $B\;S_{i}$ select it, so $n_{i}^{*}=N_{i}$ forms the PSNE.

(2) If $g_{3}\left(1\right)<A_{3,i}$, similar to the explanation in the proof of Theorem 2, $n_{i}^{*}=0$ forms the PSNE.

(3) Otherwise, $n^{*}$ satisfying Equation (33) forms the PSNE.

To evaluate the impact of ICI in CGC-FFR, we also consider a basic case where OFD between picocells and the macrocell is adopted, *i.e.*, picocells use $B_{i}$ and the macrocell uses $B_{0}-B_{i}$, called the congestion game with capacity under the condition of OFD (CGC-OFD). In this case, ICI is completely eliminated, so $g_{3}\left(n_{i}\right)$ and $A_{3,i}$ in Theorem 3 are changed into:

(34) $$g_{4}\left(n_{i}\right)=\log_{2}{\left(1+\frac{GP_{i}{\left[N_{0}/\left(n_{i}R_{0}^{2}\right)\right]}^{\gamma /2}}{\sigma^{2}}\right)}^{\frac{N_{0}-\sum_{\alpha }n_{\alpha }+1}{n_{i}}}$$

(35) $$A_{4,i}=\log_{2}{\left(1+\frac{GP_{0}/D_{0i}^{\gamma }}{\sigma^{2}}\right)}^{\frac{B_{0}-B_{i}}{B_{i}}}$$

## 5. Performance Evaluation

In this section, we provide extensive simulations by MATLAB to demonstrate the correctness of the theorems and the impact of ICI on the PSNE for the CS games with a large amount of densely-deployed devices in HCNs. The simulation area is a single macrocell with a radius equaling 1000 m. A small number of picocells are almost randomly deployed within the coverage of the macrocell, but assuming that they do not overlap with one another. This is achieved by randomly choosing the picocells’ locations one-by-one and making sure each one is not overlapped with those already deployed.

Since this study considers the uplink, the transmission power is set to 23 dBm for devices selecting the macrocell [71]. By contrast, the transmission power for the devices selecting picocells should be less than this because of power control, expanding from −9–10 dBm, adjusted to fit for different picocell sizes and path loss exponents. Each macrocell uses the total uplink bandwidth of the system, *i.e.*, 10 MHz, while picocells get part of the total uplink bandwidth because of the ICI coordination for picocells in HCNs. The AWGN spectral density is set to −174 dBm/Hz, and the radio frequency is set to 1.9 MHz. We set the density to $10^{5}$ devices/macrocell with a uniform distribution within its cell coverage. The maximum number of rounds of simulations for obtaining each point in the following figures is 100, and each round has newly deployed positions of the picocells, as well as the devices.

In each round, we use the method proposed by I. Milchtaich in [72] to search for the PSNE. The method is to check each player’s willingness to change strategy, and the searching process reaches a PSNE if no player feels like making further changes. This method guarantees the convergence to the PSNE in just a few iterations for congestion games, so it fits quite well for this study.

### 5.1. Verification of the Theorems

In this subsection, the three theorems are verified by simulations. Three picocells with radii of 100, 200 and 300 m are randomly deployed, and their PSNE are obtained by the Milchtaich method. Then, we take the PSNE into the theorems to verify their correctness.

**Figure 5:**
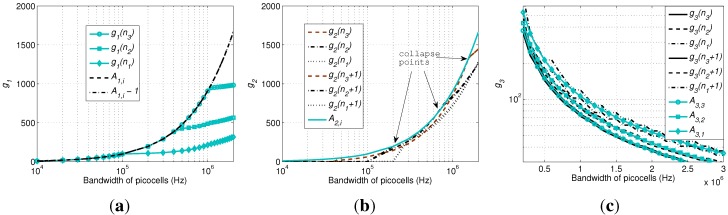
Verification of the theorems. (**a**) Theorem 1; (**b**) Theorem 2; (**c**) Theorem 3.

For Theorem 1, by increasing $B_{i}$, we can see that both $g_{1}\left(n_{i}\right)$ and $A_{1,i}$ increase, and $g_{1}\left(n_{i}\right)$ is perfectly located between $A_{1,i}-1$ and $A_{1,i}$. When $B_{i}$ is larger than a certain threshold for each cell radius, all of the devices under the coverage of this picocell select it, so the curve of $g_{1}\left(n_{i}\right)$ leaves $A_{1,i}-1$ by keeping $n_{i}^{*}=N_{i}$ for the PSNE, as shown in Figure 5a.

For Theorem 2, we set the transmission power of the devices selecting the three picocells as −9, −3 and 0.5 dBm, so that their coverage increases proportionally. The calculation of $g_{2}\left(n_{i}\right)$ is not based on the ICI obtained by Equation (12), which is unstable due to the instantaneous distribution of the devices. Instead, Equation (A15) obtained in the Appendix, as an average value, is used. Note that this average value is derived under the condition γ=2, so that a closed-form expression as an analytical result can be obtained to compare to the simulation results and to validate the theorems. Based on our experience, when *w* is relatively small, the result of CGI is quite similar to that of TCG. By contrast, when *w* is larger than a threshold, the PSNE collapses to all zeros, *i.e.*, all of the devices select the macrocell. Therefore, we choose $w=10^{6}$ to show this phenomenon in Figure 5b. We can see that $A_{2,i}$ is not within $g_{2}\left(n_{i}\right)$ and $g_{2}\left(n_{i}+1\right)$, except for the three collapse points, one for each picocell. In detail, before each collapse point (*i.e.*, on the right side), the PSNE have $n_{i}^{*}=N_{i}$. While after each collapse point (*i.e*., on the left side), the PSNE collapse to all zeros, so the curves leave $A_{2,i}$ immediately.

For Theorem 3, the same configuration as above is used. To show clearly the relationship between $g_{3}\left(n_{i}\right)$ and $A_{3,i}$, the total bandwidth of picocells (*i.e.*, the x-axis) is set to [0.2, 3] MHz. We can see that the curves for $A_{3,i}$ are smooth, while the curves for $g_{3}\left(n_{i}\right)$ and $g_{3}\left(n_{i}+1\right)$, as functions of $n_{i}$, are slightly fluctuating. ∀i∈{1,2,3}, $A_{3,i}$ is perfectly located between $g_{3}\left(n_{i}\right)$ and $g_{3}\left(n_{i}+1\right)$, indicating the correctness of Theorem 3.

### 5.2. Impact of ICI by Comparing TCG and CGI

In this subsection, we randomly deploy five picocells with identical radii in each round of simulation. For different curves in each figure, the radii of picocells vary from 100–300 m. In order to achieve different coverage radii for picocells, we set the transmission power for devices selecting picocells as −9, −3 and 0.5 dBm for picocell radii equaling 100, 200 and 300 m when the path loss exponent is two (free space). When the path loss exponent is three (suburbs), the transmission power is set to −9, 0 and 5.3 dBm, respectively. Additionally, when the path loss exponent is four (downtown), they are set to −9, 3 and 10 dBm. Because the magnitude of ICI changes along with the change of the path loss exponent, the collapse point for the weight of ICI varies for different path loss exponents.

**Figure 6:**
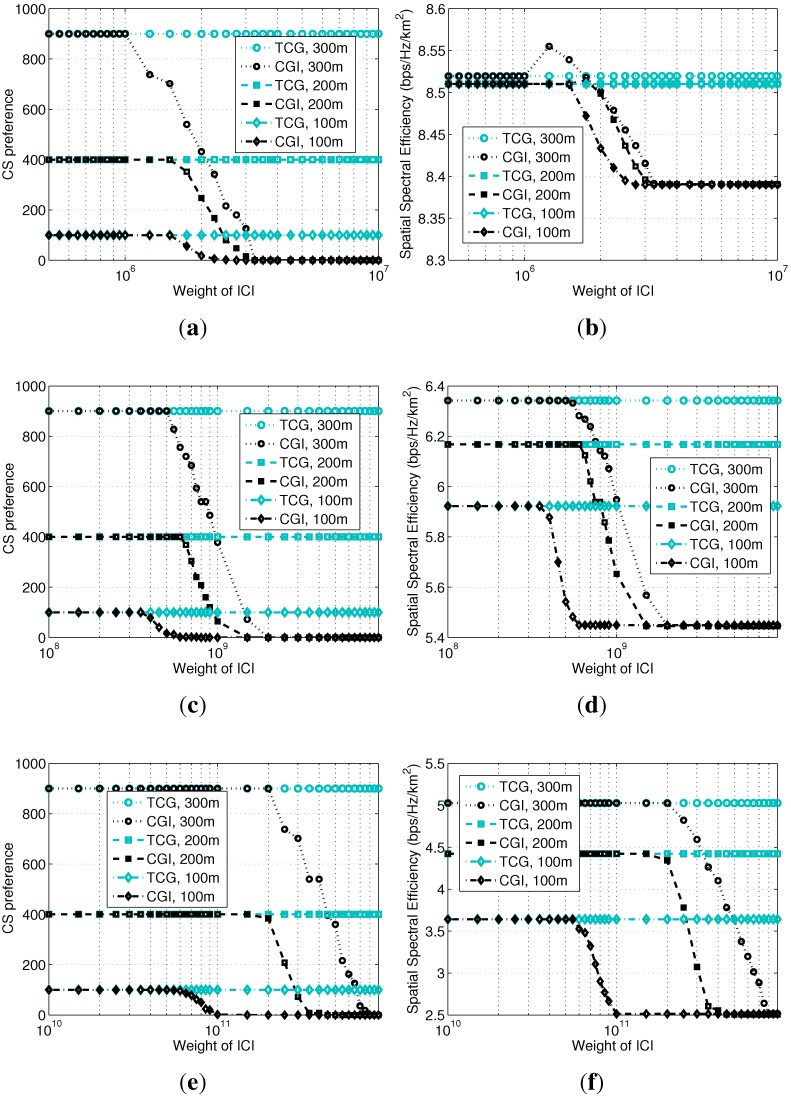
Impact of ICI on CS preference and spatial spectral efficiency using TCG and the congestion game with ICI (CGI). (**a**) Path Loss 2 (free space); (**b**) Path Loss 2 (free space); (**c**) Path Loss 3 (suburbs); (**d**) Path Loss 3 (suburbs); (**e**) Path Loss 4 (downtown); (**f**) Path Loss 4 (downtown).

We compare TCG and CGI to show the collapse in Figure 6. The x-axis is the weight of ICI, so that we could easily see the collapse of the PSNE of the CGI model and the degradation of the system performance with regard to the severity of the ICI. Note that curves for TCG in Figure 6 correspond to the PSNE, while curves for CGI do not correspond to the PSNE, but the average feature of the CGI model. As explained for Figure 4a and Figure 5b, once a device collapses to the macrocell, the PSNE collapses to all zeros without any equilibrium between $N_{i}$ and zero. Therefore, curves for CGI in Figure 6a, c and e actually show the average of $n_{i},i=1,\ldots ,5$ for a number of rounds of simulations. Since the devices’ deployment and the BSs’ locations vary round by round, the collapse points vary, as well. Actually, in each round, the collapse points for different picocells may not be the same either due to the difference of their locations. The average value of $n_{i}$ for a given cell radius and the weight of ICI can be considered as an average measure of CS preference between picocells and the macrocell. Seen from each subfigure above, the PSNE has larger probability to collapse when the weight of ICI becomes larger, indicating the impact of ICI on the PSNE. Comparing the three subfigures, when the path loss exponent is small, PSNE collapse in a relatively small weight of ICI, indicating the severity of ICI with regard to the path loss exponent.

In Figure 6b, d and f, spatial spectral efficiency with Path Losses 2, 3 and 4 are shown, respectively. The spatial spectral efficiency at each point in each curve is calculated as the summation of all of the devices’ total capacity divided by the system bandwidth and the macrocell disc area (in km${}^{2}$). We can see that the average spatial spectral efficiency decreases after the PSNE collapses. Note that, TCG does not consider the ICI at all, so its PSNE and corresponding average spatial spectral efficiency do not change along with the weight of ICI, as shown in all of the above subfigures.

As we know, for the macrocell, its advantage is the low ICI, while the disadvantage is the small available bandwidth due to the large number of connected devices. By contrast, for the picocells, the advantage is the large available bandwidth, while the disadvantage is severe ICI from the macrocell. Therefore, it is not difficult to understand the simulation results in Figure 6. Devices approaching picocell BSs obtain larger spectral efficiency by using picocells because of the larger amount of obtained bandwidth and better channel condition to these BSs. When the radii of the picocells are large, more devices will change their preferences during the collapse, so the decrement of spatial spectral efficiency will be large, which is clearly shown in all of the above subfigures. When the path loss exponent becomes larger, the difference of the channel conditions to picocells and the macrocell becomes larger, so the decrement of the spatial spectral efficiency can be very large when the PSNE collapses, which is clear by comparing Figure 6d, f with Figure 6b.

Besides, there is a small augmentation in Figure 6b before the performance collapses, this happens occasionally when the transmission power of picocell devices, the ICI and the picocell radius coincide. In detail, when the picocell radius and the ICI are both large, but the transmission power is relatively small, devices on the edge of a picocell’s coverage area should select the macrocell to guarantee system optimality.

### 5.3. Impact of ICI by Comparing CGC-FFR and CGC-OFD

The configuration for this subsection is similar to Section 5.2, except for the bandwidth and the transmission power are configured as follows. For CGC-FFR, the whole system bandwidth is used in the same way as before. By contrast, for CGC-OFD, the whole bandwidth is divided into two portions, *i.e.*, 2/3 for the macrocell and 1/3 for each picocell. The transmission powers for the picocells with 100 m radii are variable for the x-axis, from −80–20 dBm, while the transmission powers for the picocells with 200 m and 300 m radii are calculated with an augmentation as before to guarantee the increment of the picocell radii, *i.e.*, 6 and 9.5 dBm augmentation for a path loss exponent equaling two; 9 and 14.3 dBm augmentation for a path loss exponent equaling three; and 12 and 19 dBm augmentation for a path loss exponent equaling four. Note that the transmission power of a device selecting a picocell should not be as low as −80 dBm, but we keep the x-axis range large to show the collapse clearly and to compare between CGC-FFR and CGC-OFD.

**Figure 7:**
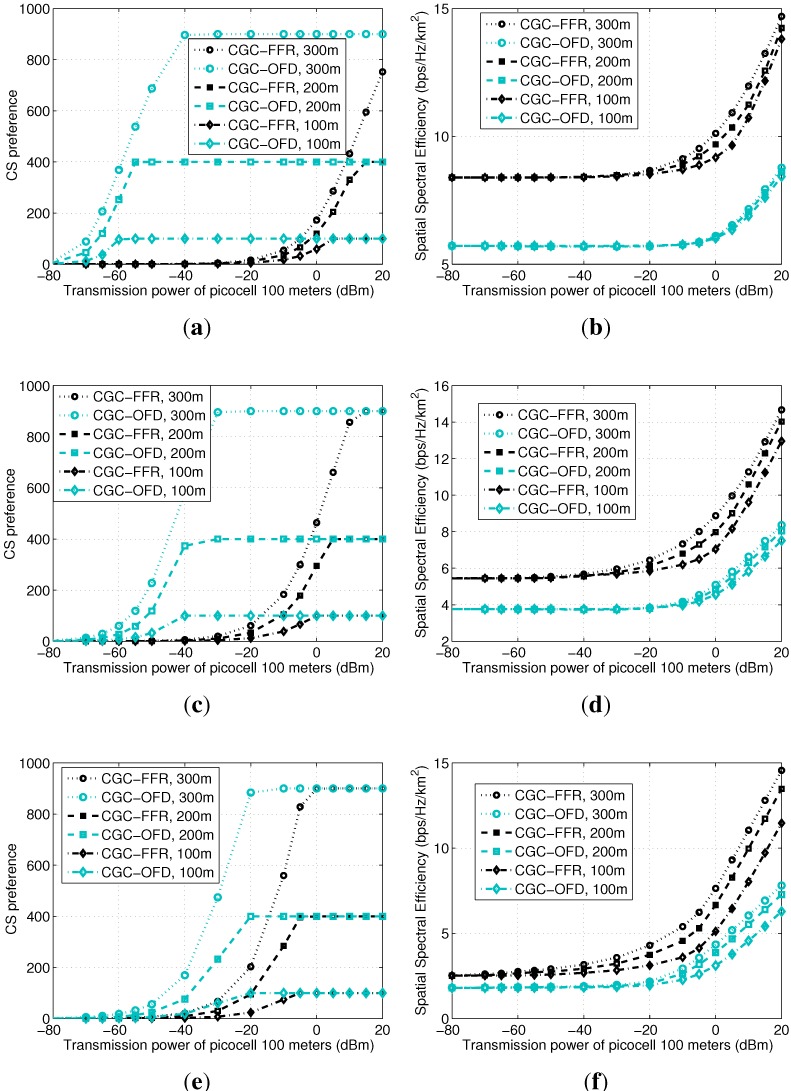
Impact of ICI on CS preference and spatial spectral efficiency using CGC-fractional frequency reuse (FFR) and CGC-orthogonal frequency division (OFD). (**a**) Path Loss 2 (free space); (**b**) Path Loss 2 (free space); (**c**) Path Loss 3 (suburbs); (**d**) Path Loss 3 (suburbs); (**e**) Path Loss 4 (downtown); (**f**) Path Loss 4 (downtown).

Figure 7a,c,e shows the collapse of the PSNE for a path loss exponent equaling 2, 3 and 4, respectively. We can see that, when the transmission power of picocells decreases, both of the two games’ PSNE collapse. The difference is that CGC-OFD keeps a number of devices selecting picocells in the normal transmission power range, while the ICI in CGC-FFR already makes the PSNE collapse to the macrocell. Besides, by increasing the path loss exponent, the gap between the curves of CGC-FFR and CGC-OFD becomes smaller. That is because the large path loss exponent decreases ICI, hence reducing the difference of the two games. Based on the analysis in Figure 4b and our experience with extensive simulations, the collapse feature of CGC is different from that of CGI. Instead of suddenly collapsing to all zeroes, the PSNE of CGC decreases gradually from $N_{i}$ to zero, little by little.

The system’s average spatial spectral efficiencies with a path loss exponent equaling 2, 3 and 4 are shown in Figure 7b,d,f, respectively. Different from the previous subfigures showing a saturated point where a curve becomes flat, curves in these three subfigures will keep on increasing along with the increase of the transmission power of picocells. We can see that, first, the curves of CGC-FFR are higher than those of CGC-OFD, indicating that, suffering severe ICI, it is still suggested to reuse the frequency. Second, when the path loss exponent is large, the difference of the spatial spectral efficiencies of the two games decreases due to the decrease of ICI. Third, the curves with larger picocells have better system performance, indicating that, seen from the system’s point of view, it is still important to keep a number of devices on the side of picocells, *i.e.*, the PSNE collapsing to the macrocell is generally an undesired result.

## 6. Conclusions

HCNs and IoT have been considered to form a new wireless network paradigm to evolve the mobile communication system toward 5G, while the CS game among densely-deployed sensor and mobile devices in HCNs became one of the key issues and challenges. For a CS game undergoing severe ICI, the congestion game model was found to be a suitable methodology for this study. In this article, we embedded ICI into the congestion game and theoretically identified the PSNE with three theorems. The closed-form expression for the ICI was calculated and helped validate the three theorems. We conclude that, on the one hand, the CS game among a large amount of densely-deployed devices can be considered from the statistical point of view by integrals, which made this game able to be solved with closed-form expressions. On the other hand, the PSNE of such a game may suddenly collapse to the macrocell under the impact of severe ICI, and the system performance, in terms of average spatial spectral efficiency, could be severely degraded by this collapse phenomenon. Therefore, this study should be quite helpful to design the future CS scheme for dense devices in an integrated environment with HCNs and IoT. Our future work involves the design of a CS scheme fitting for generalized scenarios, such as different traffic types, device types and user preferences, as well as device mobility. Meanwhile, we will theoretically consider a more generalized cell deployment, *i.e.*, the irregular deployment of BSs with Voronoi cell boundaries.

## Figures and Tables

**Figure A1:**
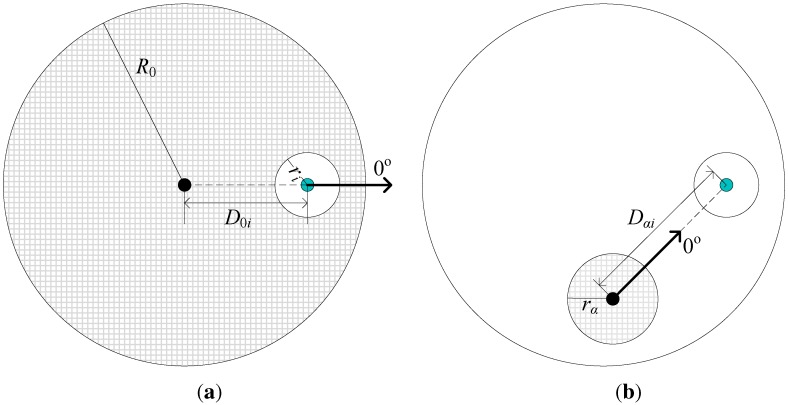
The polar coordinate systems for calculating the ICI. (**a**) To calculate *x*; (**b**) to calculate *y*.

## Appendix

Define:

(A1) $$x=\sum_{j\in D\;V_{All}}\frac{1}{d_{ij}^{2}}-\sum_{j\in D\;V_{i}}\frac{1}{d_{ij}^{2}}$$

(A2) $$y=\sum_{j\in D\;V_{\alpha }}\frac{1}{d_{\alpha j}^{2}}$$

Equation (12) can be represented by:

(A3) $$I_{0i}=\frac{GP_{0}}{N_{0}-\sum_{\alpha }n_{\alpha }}\left(x-\sum_{\alpha \neq i}y\right)$$

To obtain $I_{0i}$, we should derive *x* and *y*, respectively. For *x*, we establish the polar coordinate system, as shown in Figure A1a. The picocell $B\;S_{i}$ is exactly at the pole, while the macrocell $B\;S_{0}$ is at $\left(D_{0i},\pi \right)$. Thus, the equation of the coverage border circle for $B\;S_{0}$ and the circle formed by $D\;V_{i}$ are:

(A4) $$s^{2}+D_{0i}^{2}+2sD_{0i}\cos\theta =R_{0}^{2}$$

(A5) s=r

Equation (A4) is a quadratic equation of *s*, which can be solved by the quadratic formula as:

(A6) $$s=\sqrt{R_{0}^{2}-D_{0i}^{2}\sin^{2}\theta }-D_{0i}\cos\theta$$

Equation (A6) provides the relationship between *s* and θ. Note that the other solution is negative, which cannot be used to represent the distance.

Based on the above analysis, *x* can be rewritten as:

(A7) $$\begin{array}{cc} x & =\frac{N_{0}}{\pi R_{0}^{2}}\int_{0}^{2\pi }\int_{r_{i}}^{\sqrt{R_{0}^{2}-D_{0i}^{2}\sin^{2}\theta }-D_{0i}\cos\theta }\frac{1}{s^{2}}sdsd\theta \\ & =\frac{2N_{0}}{\pi R_{0}^{2}}\left\{\int_{0}^{\pi }\left[\ln\left(\sqrt{R_{0}^{2}-D_{0i}^{2}\sin^{2}\theta }-D_{0i}\cos\theta \right)\right]d\theta -\pi \ln r_{i}\right\} \end{array}$$

To solve the above integral, let:

(A8) $$\begin{array}{cc} z & =\int_{0}^{\pi }\ln\left(\sqrt{R_{0}^{2}-D_{0i}^{2}\sin^{2}\theta }-D_{0i}\cos\theta \right)d\theta \\ & =\int_{0}^{\pi }\ln\left[\frac{\left(\sqrt{R_{0}^{2}-D_{0i}^{2}\sin^{2}\theta }-D_{0i}\cos\theta \right)}{\left(\sqrt{R_{0}^{2}-D_{0i}^{2}\sin^{2}\theta }+D_{0i}\cos\theta \right)}\cdot \left(\sqrt{R_{0}^{2}-D_{0i}^{2}\sin^{2}\theta }+D_{0i}\cos\theta \right)\right]d\theta \\ & =\int_{0}^{\pi }\ln\left[\frac{R_{0}^{2}-D_{0i}^{2}}{\sqrt{R_{0}^{2}-D_{0i}^{2}\sin^{2}\theta }+D_{0i}\cos\theta }\right]d\theta \\ & =\pi \ln\left(R_{0}^{2}-D_{0i}^{2}\right)-\int_{0}^{\pi }\ln\left[\sqrt{R_{0}^{2}-D_{0i}^{2}\sin^{2}\theta }+D_{0i}\cos\theta \right]d\theta \end{array}$$

Let ϕ=π-θ, we find:

(A9) $$\begin{array}{cc} z & =\pi \ln\left(R_{0}^{2}-D_{0i}^{2}\right)-\int_{\pi }^{0}\ln\left[\sqrt{R_{0}^{2}-D_{0i}^{2}\sin^{2}\left(\pi -\phi \right)}+D_{0i}\cos\left(\pi -\phi \right)\right]d\left(\pi -\phi \right)\\ & =\pi \ln\left(R_{0}^{2}-D_{0i}^{2}\right)-\int_{0}^{\pi }\ln\left[\sqrt{R_{0}^{2}-D_{0i}^{2}\sin^{2}\phi }-D_{0i}\cos\phi \right]d\phi \\ & =\pi \ln(R_{0}^{2}-D_{0i}^{2})-z \end{array}$$

We obtain:

(A10) $$z=\frac{\pi }{2}\ln\left(R_{0}^{2}-D_{0i}^{2}\right)$$

Taking Equation (A10) into Equation (A7), we obtain:

(A11) $$\begin{array}{cc} x & =\frac{2N_{0}}{\pi R_{0}^{2}}\left[\frac{\pi }{2}\ln\left(R_{0}^{2}-D_{0i}^{2}\right)-\pi \ln r_{i}\right]\\ & =\frac{N_{0}}{R_{0}^{2}}\left[\ln\left(R_{0}^{2}-D_{0i}^{2}\right)-\ln\frac{R_{0}^{2}}{N_{0}}-\ln n_{i}\right] \end{array}$$

To calculate *y*, we establish the polar coordinate system as shown in Figure A1b, then:

(A12) $$y=\frac{N_{0}}{\pi R_{0}^{2}}\int_{0}^{r_{\alpha }}\int_{0}^{2\pi }\frac{s}{s^{2}+d_{\alpha j}^{2}-2sd_{\alpha j}\cos\theta }d\theta ds$$

Let $a=d_{\alpha j}^{2}+s^{2}$ and $b=-2d_{\alpha j}s$, we have $a^{2}>b^{2}$, so the above integral becomes:

(A13) $$\begin{array}{cc} y & =\frac{N_{0}}{\pi R_{0}^{2}}\int_{0}^{r_{\alpha }}s\int_{0}^{2\pi }\frac{1}{a+b\cos\theta }d\theta ds\\ & =\frac{N_{0}}{\pi R_{0}^{2}}\int_{0}^{r_{\alpha }}\frac{2s}{\sqrt{a^{2}-b^{2}}}\left[\arctan\left(\sqrt{\frac{a-b}{a+b}}\cdot \tan\frac{\theta }{2}{|}_{0}^{\pi }\right)+\arctan\left(\sqrt{\frac{a-b}{a+b}}\cdot \tan\frac{\theta }{2}{|}_{\pi }^{2\pi }\right)\right]\\ & =\frac{N_{0}}{R_{0}^{2}}\int_{0}^{r_{\alpha }}\frac{2s}{\sqrt{a^{2}-b^{2}}}ds \end{array}$$

Since we assume that the picocells do not overlap with one another, we have $D_{\alpha i}\geq s$, so:

(A14) $$\begin{array}{cc} y & =\frac{N_{0}}{R_{0}^{2}}\int_{0}^{r_{\alpha }}\frac{2s}{d_{\alpha j}^{2}-s^{2}}ds\\ & =\frac{N_{0}}{R_{0}^{2}}\left[\ln D_{\alpha i}^{2}-\ln\left(D_{\alpha i}^{2}-r_{\alpha }^{2}\right)\right] \end{array}$$

Finally, taking Equation (A11) and Equation (A14) into Equation (A3), $I_{0i}$ is obtained as a function of $n_{i}$, written as:

(A15) $$\begin{array}{cc} I_{0i}\left(n_{i}\right) & =\frac{N_{0}GP_{0}}{\left(N_{0}-\sum_{\alpha }n_{\alpha }\right)R_{0}^{2}}\left\{\left[\ln\left(R_{0}^{2}-D_{0i}^{2}\right)-\ln\frac{R_{0}^{2}}{N_{0}}-\ln n_{i}\right]-\sum_{\alpha \neq i}\left[\ln D_{\alpha i}^{2}-\ln\left(D_{\alpha i}^{2}-\frac{R_{0}^{2}n_{i}}{N_{0}}\right)\right]\right\} \end{array}$$
